# An Rv1471-expressing chimpanzee adenovirus vaccine confers protection against tuberculosis by inducing alveolar macrophage trained immunity and polyfunctional T-cell responses

**DOI:** 10.1080/22221751.2026.2637292

**Published:** 2026-02-24

**Authors:** Huiling Wang, Ying Zhang, Jianhui Li, Juan Wu, Shaoqiong Huang, Shiqi Xie, Xuejiao Huang, Jing Wang, Xiao-Yong Fan, Zhidong Hu

**Affiliations:** Shanghai Public Health Clinical Center & Shanghai Institute of Infectious Diseases and Biosecurity, Fudan University, Shanghai, People’s Republic of China

**Keywords:** Mycobacterium tuberculosis, Rv1471, trained immunity, alveolar macrophage, vaccine

## Abstract

The limited protection afforded by Bacille Calmette-Guérin (BCG) against pulmonary tuberculosis (TB) underscores the critical need for novel vaccine strategies. Alveolar macrophages (AMs), as the primary sentinel cells encountering inhaled *Mycobacterium tuberculosis* (*Mtb*), play a decisive role in early infection outcomes, yet their potential as a direct vaccine target remains largely untapped. Here, we developed a chimpanzee adenovirus vaccine expressing the *Mtb* antigen Rv1471 (rAd-Rv1471), which we previously identified for its unique capacity to induce innate immune memory. In murine models, intranasal rAd-Rv1471 administration reprogrammed AMs into a trained state, characterized by enhanced production of pro-inflammatory cytokines, elevated surface expression of MHC II and CD86, and improved cell-intrinsic control of intracellular mycobacterial growth. Transcriptomic analysis revealed upregulation of key immunometabolic pathways, including Akt/mTOR/HIF-1α signalling and glycolysis. Concurrently, intranasal rAd-Rv1471 administration induced potent antigen-specific, polyfunctional T cells in the lung. This dual engagement of innate and adaptive immunity conferred significant protection against aerosol *Mtb* challenge. Furthermore, rAd-Rv1471 acted as an effective heterologous booster, enhancing protection in BCG-primed mice. Our findings establish rAd-Rv1471 as a synergistic mucosal vaccine candidate that concurrently induces trained immunity in AMs and polyfunctional T-cell responses, highlighting a promising dual-targeting strategy for next-generation TB vaccines.

## GRAPHICAL ABSTRACT

**Figure:**
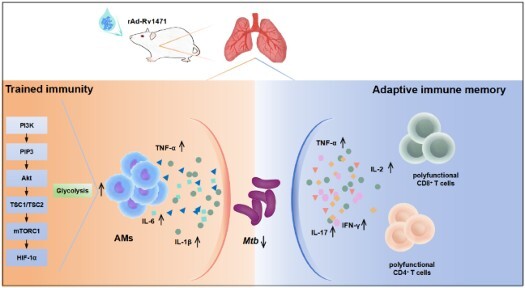

## Introduction

Tuberculosis (TB), a chronic infectious disease caused by *Mycobacterium tuberculosis* (*Mtb*), remains the deadliest single pathogen-induced disease globally, accounting for 1.25 million deaths in 2023 [1]. Currently, the only licensed TB vaccine is the century-old Bacille Calmette-Guérin (BCG), with over four billion doses administered worldwide [2]. Although BCG elicits robust Th1 immune responses and demonstrates strong protective efficacy in children, its effectiveness in adolescents and adults is highly variable (0%−80%); repeated vaccinations fail to enhance protection, limiting its efficacy in controlling the global TB epidemic [3,4]. One proposed explanation for BCG’s suboptimal efficacy is its inability to generate sufficient lung tissue-resident memory T cells (T_RM_) via systemic immunization. To address this, we previously developed a recombinant Sendai virus-vectored TB vaccine that induced robust lung CD8^+^ T_RM_ in murine models, as confirmed by intravascular staining [5]. Subsequent studies reported other vaccines capable of inducing lung T_RM_ [6–9]. However, although these subunit vaccines substantially reduced bacterial loads post-infection in mice, sterilizing immunity was not achieved, highlighting the need for novel strategies to optimize the current TB vaccines.

Recent studies of “TB resisters”, individuals with repeated household exposure to TB patients who remain persistently uninfected, revealed a critical crosstalk between T-cell immunity and alveolar macrophages (AMs)-mediated innate immunity in their pulmonary alveoli [10]. Upon *Mtb* infection, IFN-γ-primed AMs in TB resisters mounted enhanced TNF responses, triggering cellular stress and promoting poly-cytotoxic T cells, this enabled efficient recognition and elimination of *Mtb*-infected AMs in a timely manner, resulting in sterilizing immunity [10]. As the first cells to phagocytize inhaled *Mtb* [11], AMs play a pivotal role in determining infection outcomes. Accumulating evidence suggests that the early interplay between AMs and memory T cells may control or even clear the invading *Mtb* [11–15]. Nevertheless, whether AMs-mediated innate immunity can be harnessed by novel TB vaccines to optimize immune protection remains unclear.

Immune memory was long considered exclusive to adaptive immunity. However, the concept of “trained immunity” (innate immune memory) proposes that innate immune cells can be functionally reprogrammed by exogenous/endogenous stimuli; following return to a non-activated state, these cells exhibit altered proinflammatory responses that lead to enhanced immune protection against secondary homologous/heterologous infection [16–18], We therefore hypothesized whether novel TB vaccines could induce innate immune memory in AMs. For this purpose, we selected a chimpanzee adenovirus vector, since it was recently reported that adenoviral vectors of human and chimpanzee origin could induce autonomous memory AMs in mice [19,20]. Additionally, we chose the *Mtb* antigen Rv1471, which we previously identified as the first single *Mtb* antigen capable of inducing innate immune memory in macrophages and conferring immune protection against *Mtb* infection as a protein subunit vaccine in murine models [21].

Herein, we constructed a novel replication-defective chimpanzee adenovirus expressing Rv1471, rAd-Rv1471. We hypothesized that this vaccine would enhance trained immunity in AMs, thereby synergizing with polyfunctional T-cell responses to strengthen anti-TB protection. Using comprehensive *in vitro* and *in vivo* approaches, we systematically evaluated rAd-Rv1471’s ability to induce AM-trained immunity, elicit antigen-specific T-cell responses, and confer protection against *Mtb* challenge in mice. This study proposes a novel TB vaccine strategy focused on modulating AM-trained immunity. Unlike approaches that primarily aim to boost adaptive memory, our strategy seeks to simultaneously engage both innate and adaptive immune memory, aiming to achieve a dual-axis enhancement of protective efficacy.

## Results

### Construction and validation of rAd-Rv1471

**Figure 1.**
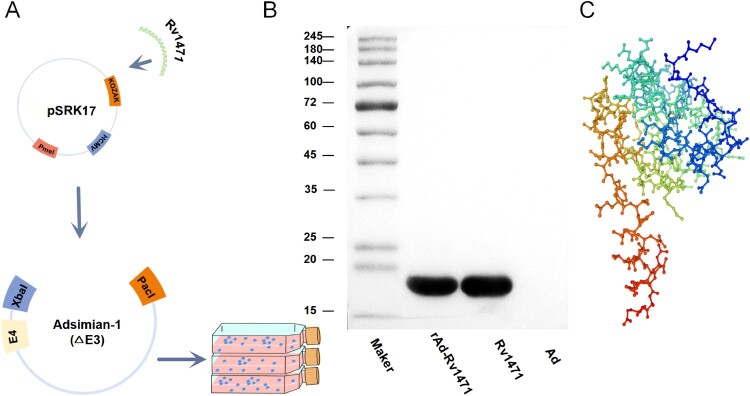
Construction and validation of rAd-Rv1471 (A) Schematic representation of rAd-Rv1471 recombinant adenovirus vaccine construction. The *Rv1471* gene was amplified from the *Mtb*-H37Rv genome by PCR and cloned into the pSRK17 shuttle plasmid. Homologous recombination integrated the gene into the Adsimian-1 (ΔE3) vector. Following *Pac I* restriction enzyme linearization, the recombinant plasmid was transfected into HEK 293A cells for viral amplification. (**B**) Western blot analysis of HEK 293A cells infected with rAd-Rv1471 revealed a specific band at approximately 15-20 kDa, consistent with the expected molecular weight of Rv1471 (13.8 kDa), which typically migrates higher due to its high isoelectric point (pI = 10.26). Purified recombinant Rv1471 protein expressed in *E. coli* TOP10 and lysates from blank Ad vector-infected cells served as positive and negative controls, respectively. (**C**) Predicted three-dimensional structure of the Rv1471 protein using SWISS-MODEL (A0A5Q5CFW0.1.A, https://swissmodel.expasy.org/interactive/j66bSm/models/).

The DNA sequence encoding the *Mtb* H37Rv antigen Rv1471 was cloned into a replication-deficient chimpanzee adenovirus type 26 vector, hereafter referred to as Ad. Expression of the Rv1471 gene is driven by an early macrophage-specific human cytomegalovirus promoter integrated into the pSK17 shuttle plasmid (Figure 1A). Sanger sequencing confirmed correct recombinant adenovirus assembly (Figure S1). Following restriction enzyme digestion, the linearized adenoviral genome was transfected into HEK 293A cells for viral packaging. Cytoplasmic extracts from infected HEK 293A cells were harvested post-propagation, purified via ultracentrifugation to remove high-molecular-weight contaminants, and validated for Rv1471 expression using anti-Rv1471 serum in Western blot analysis (Figure 1B). The secondary structure of Rv1471 was predicted using the SWISS-MODEL server (Figure 1C).

### Intranasal rAd-Rv1471 induces trained immunity in AMs

**Figure 2.**
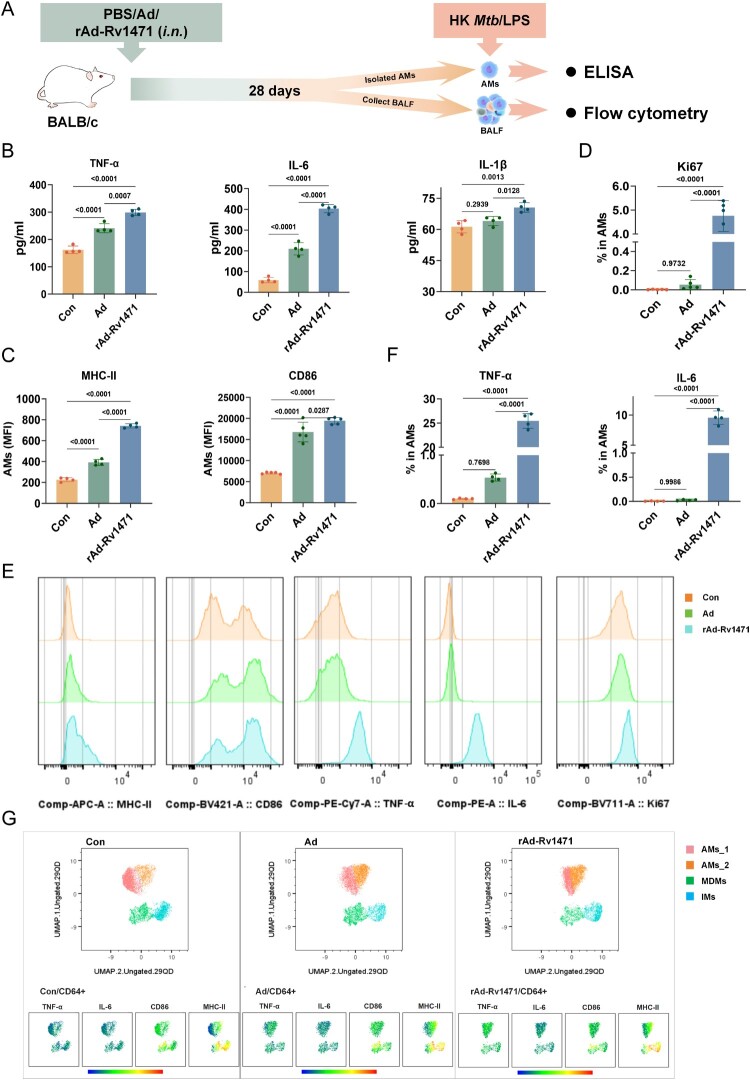
Intranasal rAd-Rv1471 training induces trained immunity in AMs. (A) Schematic representation of the mouse trained immunity evaluation model. Mice were intranasally received PBS, Ad, or rAd-Rv1471, followed by a 28-day resting period prior to AMs isolation for immunological analysis. (B) ELISA quantification of TNF-α, IL-6, and IL-1β levels in the culture supernatants following HK *Mtb* stimulation. (n = 4; one-way ANOVA). (C-G) Flow cytometric analysis on immune-trained AMs (n = 4; one-way ANOVA). (C) The mean fluorescence intensity (MFI) of MHC II and CD86 on AMs. (D) The expression of Ki67 in AMs. (E) Representative flow cytometric plots of MHC II/CD86 expression and TNF-α/IL-6 secretion in AMs. (F) The secretion levels of TNF-α/IL-6 in AMs. (G) A UMAP-based dimensionality reduction analysis was performed on CD64^+^ cells 28 days after training. Data represent two independent representative experiments and are presented as mean ± SD.

A murine model of *in vivo* trained immunity induction was employed [20,22]. As shown in Figure 2A, the AMs were isolated 28 days after intranasal training with PBS, Ad vector, or rAd-Rv1471, then restimulated with heat-killed *Mtb* H37Rv strain (HK *Mtb*) or lipopolysaccharide (LPS). Consistent with prior reports [20], enzyme-linked immunosorbent assay (ELISA) showed that Ad vector intranasal training significantly enhanced pro-inflammatory cytokines TNF-α and IL-6 secretion, and modestly increased IL-1β production upon HK *Mtb* (Figure 2B) and LPS stimulation (**Figure S2A**). Notably, rAd-Rv1471 training further amplified the secretion of TNF-α, IL-6, and IL-1β compared with Ad training upon HK *Mtb* (Figure 2B) and LPS stimulation (**Figure S2A**). These data suggest *in vivo* Rv1471-trained AMs mounted high levels of inflammatory responses against homologous/heterologous stimulation.

Flow cytometry analysis of immune cells identified AMs (CD45 ^+^ CD3^−^Ly6G^−^CD11c ^+^ CD64 ^+^ Ly6C^−^ Siglec-F^+^) and other pulmonary immune cell populations such as interstitial macrophages (IMs; CD45 ^+^ CD3^−^ Ly6G^−^CD11c ^+^ CD64 ^+^ Ly6C^−^Siglec-F^−^), monocyte-derived macrophages (MDMs; CD45 ^+^ CD3^−^Ly6G^−^CD11c^+^ CD64 ^+^ Ly6C ^+^ Siglec-F^−^), neutrophils (CD45 ^+^ CD3^−^Ly6G^+^), and T cells (CD45 ^+^ CD3^+^) in bronchoalveolar lavage fluid (BALF) [20,23], using a gating strategy shown in **Figure S2B**. Pulmonary IMs, MDMs, neutrophils, and T cells transiently increased at day 7 post-training, but returned to baseline by day 28, conversely, total macrophages and AMs decreased transiently at day 7 but fully recovered by day 28 (**Figure S2C**). Serum inflammatory cytokines also increased at day 7 and normalized by day 28 (**Figure S2D**). These data suggest that the trained cells were returned to a non-activated state at day 28.

To characterize the functional phenotypes of rAd-Rv1471-trained AMs, the expression of MHC-II (associated with trained immunity induction [20,24,25]), M1 marker (CD86), M2 marker (CD206), and cell proliferation marker (Ki67) were evaluated post HK *Mtb* stimulation. Ad training significantly upregulated MHC II, and CD86 expression in AMs, and rAd-Rv1471 training further elevated MHC-II and Ki67 expression, while CD86 expression remained comparable to that observed with Ad training (Figure 2C-E). In contrast, the surface expression of M2 marker CD206 was identical among groups (**Figure S3A**). In addition, flow cytometric analysis confirmed the secretion levels of TNF-α and IL-6 in AMs were significantly increased in the rAd-Rv1471 training group versus both Ad and PBS groups (Figure 2E-F). Further, UMAP-based dimensionality reduction analysis was performed on CD64^+^ cells (macrophages) 28 days after training. Cell populations identified AMs_1 (MHC-II ^–^ ), AMs_2 (MHC-II^+^), IMs, and MDMs. Notably, AMs in rAd-Rv1471 training group exhibited significantly higher levels of MHC-II and TNF-α (Figure 2G).

Similarly, we characterized other macrophage populations in the airway and observed that both IMs and MDMs exhibited training responses analogous to those seen in AMs following rAd-Rv1471 training (**Figure S3B-E**). In addition, to confirm Rv1471-specific effects, a control adenovirus expressing another *Mtb* antigen Rv2299c (rAd-Rv2299c) was generated. Unlike rAd-Rv1471, rAd-Rv2299c failed to enhance AMs’ trained immunity versus the Ad vector (Figure S4). Similar observations were also confirmed using other *Mtb* antigens (data not shown).

Taken together, these data corroborate prior findings that intranasal training with Ad induces trained immunity in murine models [20]. Critically, we demonstrate that adenovirus-vectored Rv1471, but not other *Mtb* antigens, potently enhanced trained immunity in airway macrophages.

### rAd-Rv1471 enhances cell-autonomous anti-mycobacterial activity in AMs

**Figure 3.**
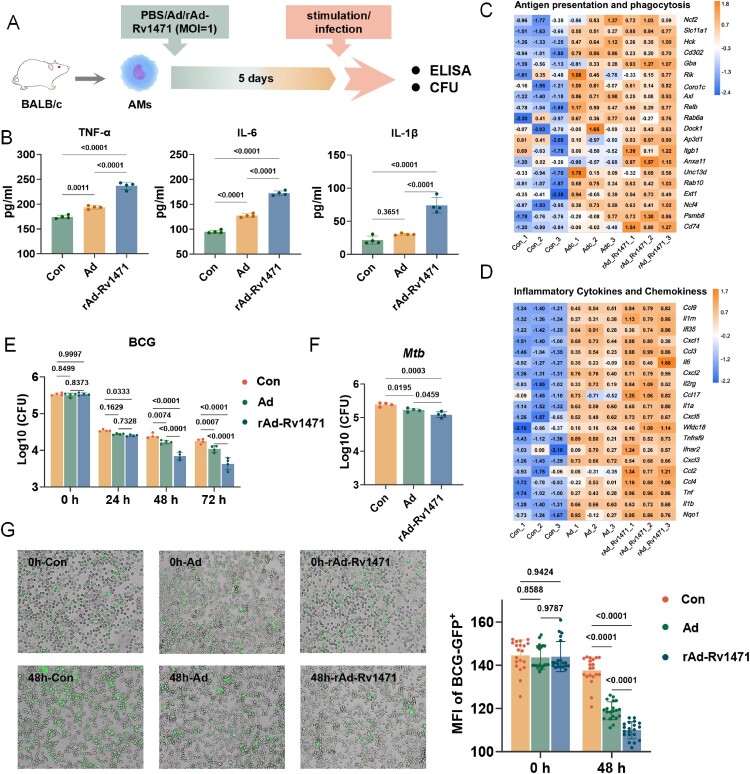
rAd-Rv1471 enhances cell-autonomous anti-mycobacterial activity in AMs. (A) Schematic representation of the *in vitro* AMs training model. Isolated AMs were cultured *in vitro* to reach appropriate confluence and then trained with PBS, Ad, or rAd-Rv1471. Analyses were performed after a 5-day resting period. (B) ELISA quantification of TNF-α, IL-6, and IL-1β in the culture supernatants following HK *Mtb* re-stimulation (n = 4; one-way ANOVA). (C-D) Heatmaps showing the expression levels of cytokines, chemokines, and key antigen-presenting genes (n = 3). (E-F) Resting AMs were infected with BCG or *Mtb* H37Rv, and bacterial loads were quantified by CFU assay (MOI = 5, n = 4; E: two-way ANOVA; F: one-way ANOVA). (G) Fluorescence microscopy analysis of BCG-GFP-infected AMs (MOI = 5). The fluorescence intensity of GFP^+^ signal from 20 fields/group at 0 and 48 h were quantified using ImageJ software, represents the MFI within the fields and correlates with the density of fluorescent bacteria (two-way ANOVA). Data represent two independent representative experiments and are presented as mean ± SD.

To determine whether rAd-Rv1471-induced trained immunity in AMs is dependent on other immune cells, an *in vitro* expanded AMs trained immunity evaluation model was utilized (Figure 3A) [26–28]. Briefly, AMs from BALF of naïve BALB/c mice were isolated and expanded *in vitro*. The immunostimulatory effects of rAd-Rv1471 were evaluated across a range of multiplicities of infection (MOI; 0.01, 0.1, 1, and 10). It was found that a MOI of 1 effectively induced a robust trained immunity response (**Figure S5A**). Therefore, this dosage was selected for all subsequent experiments. After 5 days of rest (returned to baseline; **Figure S5B-C)**, AMs were restimulated with LPS or HK *Mtb*. Mirroring our *in vivo* observations, Ad *in vitro* training exhibited significantly enhanced TNF-α and IL-6 secretion, and modestly elevated IL-1β production following HK *Mtb* and LPS stimulation. Critically, rAd-Rv1471 *in vitro* training further amplified cytokine secretion versus Ad training upon both HK *Mtb* (Figure 3B) and LPS stimulation (**Figure S5D**). Transcriptional profiling showed that rAd-Rv1471-trained AMs exhibited upregulated expression of genes encoding inflammatory cytokines (*Tnf*, *Il6*, *Il1b*, etc), chemokines (*Ccl3*, *Ccl2*, *Cxcl5*, etc), and key molecules involved in antigen presentation and phagocytosis (*Ncl2*, *Gba*, *Anxa11*, *Cd74*, etc) (Figure 3C-D). These findings indicate rAd-Rv1471 *in vitro* training reprogrammed AMs toward a pro-inflammatory state.

To directly assess whether rAd-Rv1471-induced AMs trained immunity would enhance the anti-mycobacterial efficacy, trained and naïve AMs were infected with live BCG and *Mtb* at a MOI of 5. Bacterial loads were quantified at different time points after infection. We observed significantly reduced intracellular bacterial loads in both BCG (Figure 3E) and *Mtb* (Figure 3F) in Ad-trained AMs at 48 h post-infection compared to untrained controls, with rAd-Rv1471-trained AMs exhibiting higher levels of bacterial clearance (Figure 3E-F). Further, the trained AMs were infected with BCG-GFP (MOI = 5), and it was shown that the fluorescence intensity of GFP^+^ signal was significantly lower in the rAd-Rv1471 group at 24 h (Figure 3G). Taken together, these data demonstrate rAd-Rv1471 enhanced cell-intrinsic anti-mycobacterial defenses via trained immunity, which was independent of other cells, at least in this *in vitro* model.

### Transcriptional profiles underlie rAd-Rv1471-induced trained immunity

**Figure 4.**
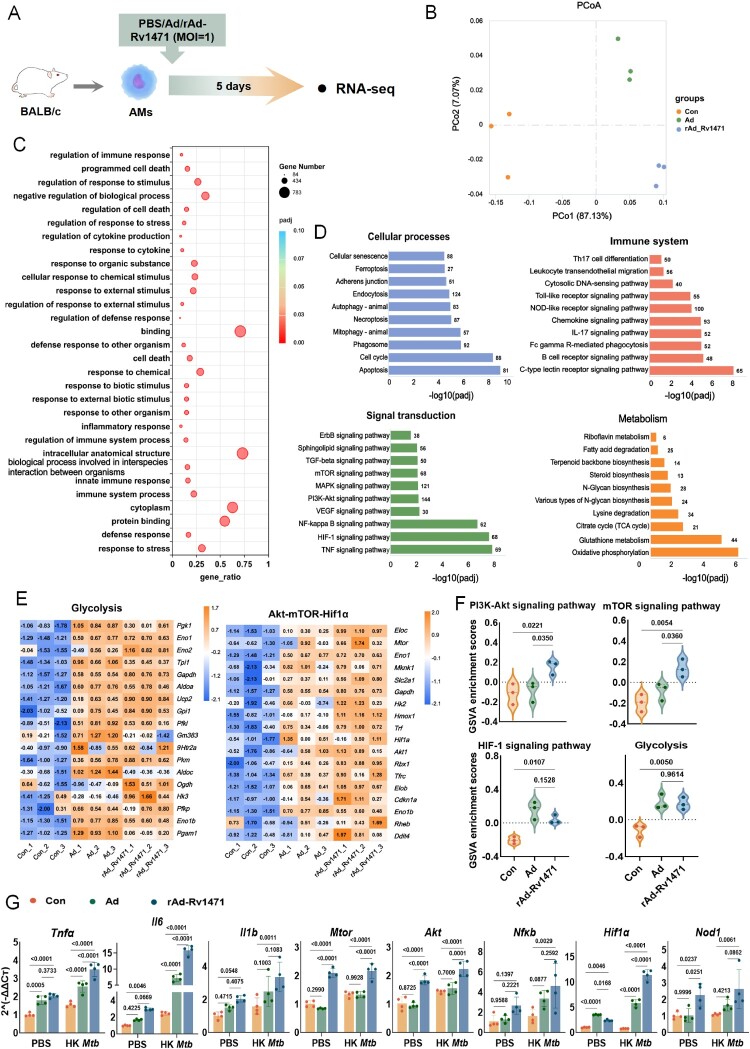
Transcriptional profiling of rAd-Rv1471-induced trained immunity. (A) Schematic representation of the experimental workflow. Sorted AMs were subjected to Smart RNA-seq on day 5 post-training. (B) PCoA of AMs. (C) GOBP enrichment analysis of differentially expressed genes. (D) KEGG pathway enrichment analysis of differentially expressed genes. (E) Heatmaps of key genes expression in glycolysis and the Akt-mTOR-HIFα signalling pathways. (F) Gene Set Variation Analysis (GSVA) violin plots showing enrichment scores of key signalling pathways, including PI3K-Akt, mTOR and HIF-1, as well as glycolysis – related gene sets (n = 3; one-way ANOVA). (G) qPCR analysis of *Tnfα, Il6, Il1b, Mtor, Akt, Nfκb, Hif1α,* and *Nod1* gene expression (n = 4; two-way ANOVA). Data represent two independent representative experiments and are presented as mean ± SD.

To delineate molecular mechanisms, we performed Smart-seq2 RNA sequencing (RNA-seq) on *in vitro*-trained AMs after 5 days of rest (Figure 4A). Principal coordinates analysis (PCoA) (Figure 4B) and differentially expressed genes (**Figure S6A and Table S1**) revealed distinct transcriptional profiles. Gene Ontology Biological Process (GOBP) enrichment analysis indicated that the upregulated pathways in rAd-Rv1471-trained AMs were primarily associated with stimulus response and innate immune response processes, whereas pathways involved in sensory processing and DNA repair were downregulated **(**Figure 4C**, Figure S6B)**, suggesting a functional reprogramming of cellular activities to facilitate more efficient gene expression during infection. Kyoto Encyclopedia of Genes and Genomes (KEGG) pathway analysis further revealed significant enrichment in classical inflammatory signalling pathways, including ErbB, TGF-β, MAPK, PI3K-Akt, NF-κB, and TNF signalling pathways, as well as pathways related to the innate immune response, such as leukocyte transendothelial migration, Toll-like receptor signalling, NOD-like receptor signalling, chemokine signalling, and C-type lectin receptor signalling (Figure 4D). Crucially, pathways central to trained immunity induction in macrophages, such as glycolysis and Akt/mTOR/HIF-1α signalling pathways were significantly activated in Ad and rAd-Rv1471 groups (Figure 4E-F), as well as linoleic acid metabolism and autophagy (**Figure S6C**), compared with the untrained group. The rAd-Rv1471 group exhibited similar glycolysis levels but significantly higher levels of Akt/mTOR/HIF-1α signalling pathway compared with the Ad group (Figure 4E-F). Furthermore, metabolic profiling using the Seahorse XF analyzer, including measurements of basal glycolysis and compensatory glycolysis, indicated that both rAd-Rv1471 and Ad exerted comparable effects on enhancing glycolytic activity (**Figure S6D**). To validate the RNA-seq analysis, qPCR was used to confirm the transcriptional levels of key genes. It was shown that expression levels of *Tnfa, Il6, Il1b, Mtor, Akt, Nfkb, Hif1a, and Nod1* were all elevated in the rAd-Rv1471 group (Figure 4G). In summary, these results demonstrate rAd-Rv1471 establishes a trained state in AMs through transcriptional reprogramming, enhancing immunometabolic responses to secondary challenges.

### rAd-Rv1471 induces robust antigen-specific T-cell immune responses

**Figure 5.**
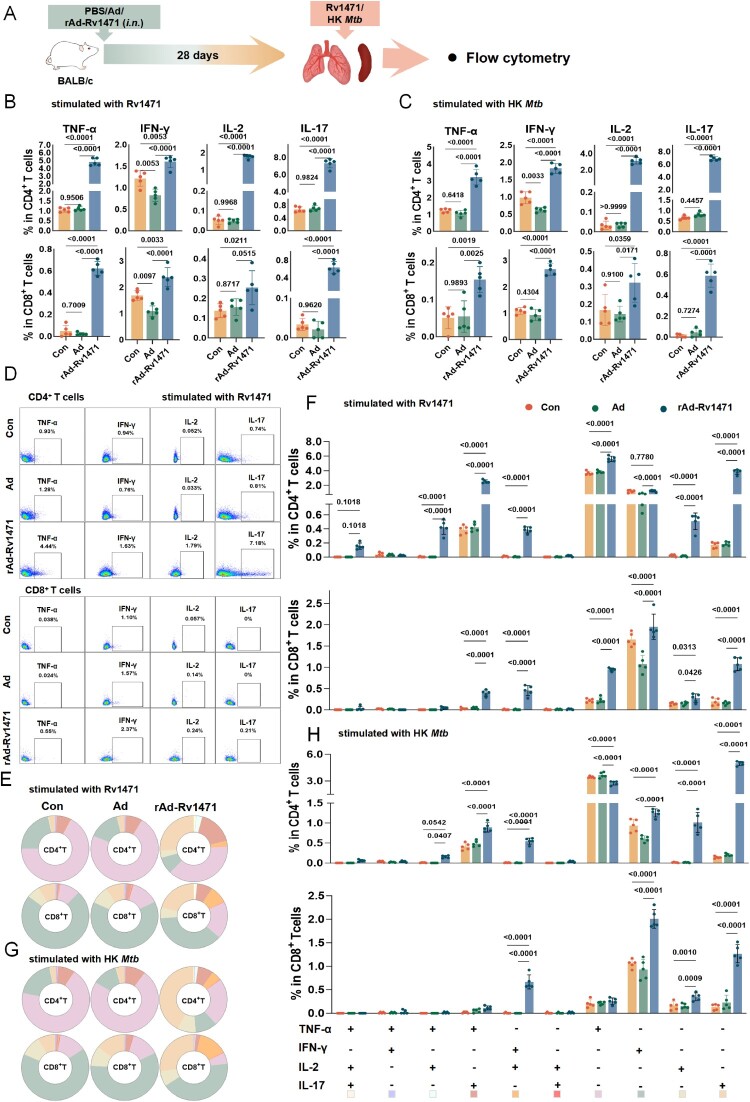
rAd-Rv1471 induces robust antigen-specific T-cell immune responses. (A) Schematic representation of the experimental workflow. (B-C) Flow cytometric analysis of the T-cell immune response in the lungs four weeks post-training with PBS, Ad, or rAd-Rv1471, following stimulation with Rv1471 (B) or HK *Mtb* (C) (n = 5; one-way ANOVA). (D) Representative flow cytometric plots depicting the expression of four intracellular cytokines. (E-H) Proportions (E) and frequencies (F) of Rv1471-specific polyfunctional CD4^+^ and CD8^+^ T cells; as well as proportions (G) and frequencies (H) of polyfunctional CD4^+^ and CD8^+^ T-cell immune responses following HK *Mtb* stimulation (n = 5; two-way ANOVA). Data represent two independent representative experiments and are presented as mean ± SD.

We further tested the immunogenicity of rAd-Rv1471 as a vaccine in murine models. BALB/c mice were intranasal trained with PBS, Ad, or rAd-Rv1471, and single-cell suspensions from the lungs and spleen were analyzed after 28 days (Figure 5A). Flow cytometric analysis (the gating strategy was shown in **Figure S7A**) revealed that mice trained with rAd-Rv1471 significantly increased frequencies of Rv1471-specific Th1 and Th17 CD4^+^ T cells, as well as cytotoxic CD8^+^ T-cell subsets (Figure 5B-D), which was reflected by enhanced secretion of TNF-α, IFN-γ, IL-2, and IL-17 in both CD4^+^ and CD8^+^ T-cell populations after Rv1471 stimulation (Figure 5B,D). Similar trends were also observed after HK *Mtb* stimulation (Figure 5C). Polyfunctional analysis further demonstrated an expansion of double-positive (TNF-α^+^IL-2^+^, TNF-α^+^IL-17^+^, IFN-γ^+^IL-17^+^), and single-positive (TNF-α^+^, IFN-γ^+^, IL-2^+^, IL-17^+^) CD4^+^ T-cell subsets that were increased in rAd-Rv1471-trained lung (Figure 5E-F). Similarly, CD8^+^ T cells displayed increased proportions of polyfunctionality (Figure 5E-F). Similar trends occurred with HK *Mtb* stimulation (Figure 5G-H), and splenic responses mirrored lung immunogenicity (Figure S8). Collectively, these findings demonstrate that the rAd-Rv1471 vaccine elicits potent antigen-specific polyfunctional T-cell responses in the lung and spleen in a murine vaccination model.

### rAd-Rv1471 confers protection against *Mtb* infection in mice

**Figure 6.**
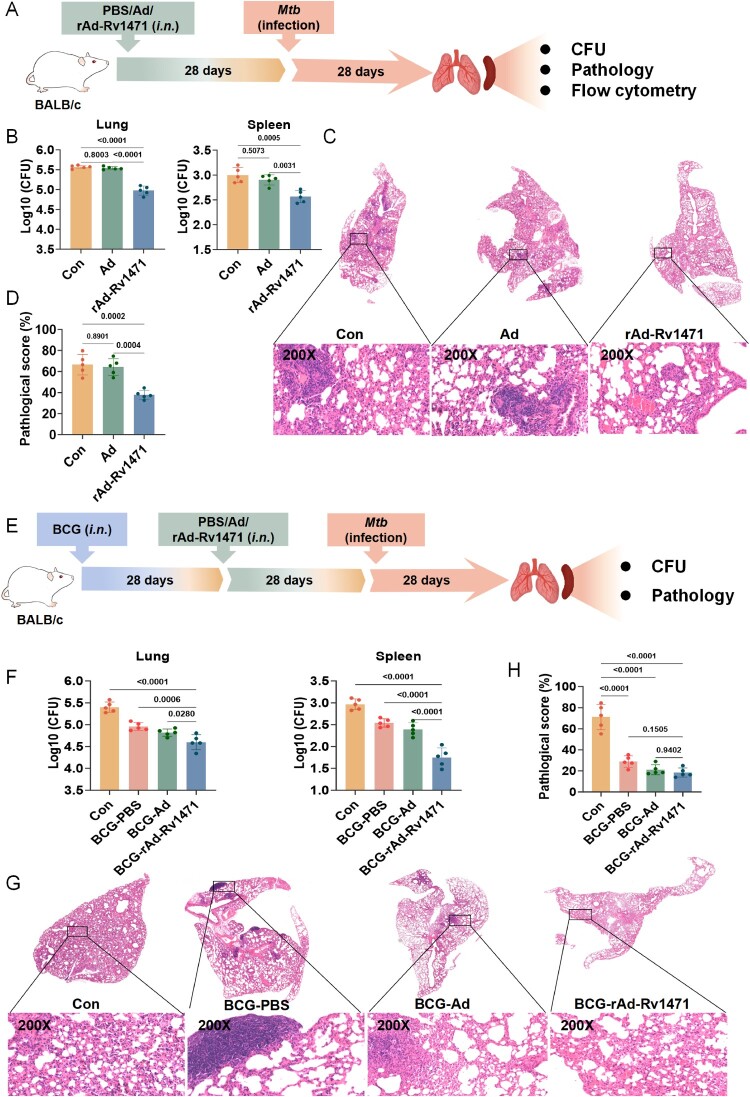
rAd-Rv1471 confers protection against *Mtb* infection in mice. (A) Schematic representation of the experimental workflow. Mice were aerosol-challenged with *Mtb* 4 weeks post-training and euthanized after 4 weeks. (B) Bacterial loads in lung and spleen homogenates were quantified by CFU counting. (C-D) Histopathology of the accessory lung lobe. Representative H&E-stained sections (Scale bars: 100 μm, ×200 magnification, top; 50 μm, ×400 magnification, bottom) were shown in (C), and pathological score calculated as the percentage of inflammatory infiltration area relative to total pleural area is shown in (D). (E) Schematic of the experimental workflow. BCG-vaccinated mice were intranasally received PBS, Ad, or rAd-Rv1471, 4 weeks post-vaccination, the mice were aerosol-challenged with *Mtb* and euthanized 4 weeks later. (F) Bacterial loads in lung and spleen homogenates were quantified by CFU counting. (G-H) Histopathology of the accessory lung lobe. Data represent two independent representative experiments (n = 5; one-way ANOVA), and are presented as mean ± SD.

To directly assess the protective efficacy against *Mtb* infection, trained mice were challenged with *Mtb* 4 weeks after vaccination (Figure 6A). The rAd-Rv1471-vaccinated mice exhibited significantly lower bacterial loads in both lung and spleen compared to Ad-trained mice, with Ad only slightly decreasing the bacterial loads in the spleen compared with non-trained mice (Figure 6B). Histopathological analysis of lung tissues identified distinct inflammatory pathologies across all groups, characterized by lymphocytes, macrophages, and granulocytes infiltration; alveolar wall thickening; and the presence of hemorrhagic foci. Notably, rAd-Rv1471-trained mice displayed a markedly amelioration of these histopathological changes (Figure 6C). Quantitative assessment of inflammatory infiltration further supported the protective effect of rAd-Rv1471 vaccination (Figure 6D). Given the limited efficacy of BCG revaccination in adults, we further assessed rAd-Rv1471 as a heterologous booster administered 4 weeks after BCG priming (Figure 6E). This vaccination strategy led to additional reductions in bacterial loads following *Mtb* challenge compared to BCG priming alone (Figure 6F). Although a reduction in inflammatory infiltration was observed, it did not reach statistical significance versus the BCG-primed group (Figure 6G-H). Collectively, these findings demonstrate that rAd-Rv1471 is effective both as a standalone vaccine and as a BCG booster to enhance anti-TB protection in mice.

### rAd-Rv1471 enhances recall T-cell responses post *Mtb* infection

**Figure 7.**
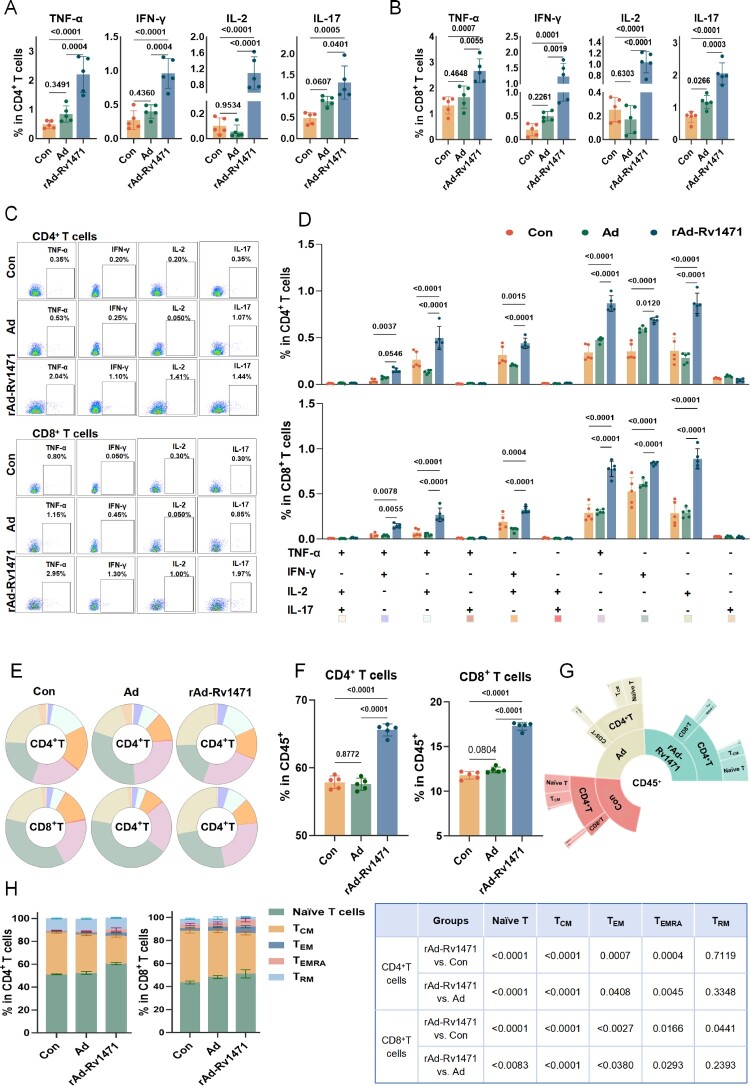
rAd-Rv1471 enhances recall T-cell responses post *Mtb* infection. (A-B) Flow cytometric analysis of cytokine production in lung-derived CD4^+^ and CD8^+^ T cells from *Mtb*-infected mice after PPD stimulation. (C) Representative flow cytometric plots of the four cytokines. (D-E) polyfunctional T-cell analysis. (D) Frequencies of PPD-specific polyfunctional CD4^+^ and CD8^+^ T cells. (E) Proportions of corresponding polyfunctional subsets. (F-H) T-cell subsets distribution. (F) Proportions of lung CD4^+^ and CD8^+^ T cells. (H) Proportions of naïve T cells, T_CM_, T_EM_, T_EMRA_, and T_RM_ (n = 5; two-way ANOVA). (G) Sunburst plot visualization of subsets. Data represent two independent experiments and are presented as the mean ± SD.

Recall immunity was assessed post-*Mtb* challenge. rAd-Rv1471-trained mice exhibited elevated levels of TNF-α, IFN-γ, IL-2, and IL-17 production in both CD4^+^ and CD8^+^ T cells compared to PBS- and Ad-trained controls (Figure 7A-C, the gating strategy was shown in Figure S7). Polyfunctional profiling demonstrated an expansion of double-positive CD4^+^ T-cell subsets (TNF-α^+^IFN-γ^+^, TNF-α^+^IL-2^+^, IFN-γ^+^IL-2^+^) and single-positive subsets (TNF-α^+^, IFN-γ^+^, IL-2^+^), with parallel increases in corresponding double- and single-positive CD8^+^ T-cell populations (Figure 7D-E). Notably, a significant increase in the proportions of CD4^+^ and CD8^+^ T cells was observed in the rAd-Rv1471-trained lung after infection (Figure 7F). Furthermore, rAd-Rv1471 training was associated with reduced frequencies of central memory T cells (T_CM_) but increased proportions of effector memory (T_EM_), terminally differentiated effector memory (T_EMRA_), and naïve T-cell subsets within both CD4^+^ and CD8^+^ T-cell populations (Figure 7G-H). We thus hypothesize that rAd-Rv1471 primed lung T cells for rapid, polyfunctional recall responses against *Mtb* infection, potentially through preferential differentiation toward effector phenotypes.

## Discussion

TB persists as the leading cause of death from a single infectious agent. As the only licensed TB vaccine, BCG provides limited protection in adults. The discrepancy between widespread infection and suboptimal efficacy of existing vaccines underscores an urgent need for novel TB vaccine strategies. Recent advances reveal that innate immune cells develop long-term functional reprogramming, characterized by metabolic and epigenetic remodeling that enhances rapid and broad-spectrum protective responses to heterologous challenges, a phenomenon termed trained immunity [16–18,29]. Numerous studies have shown that trained macrophages exhibit heightened inflammatory responses, alongside enhanced phagocytic and bactericidal capacities, improving host defense against bacterial infections [30,31], viral infections [32,33], and tumour invasion [34,35]. This paradigm offers transformative potential for next-generation TB vaccines.

Our prior work identified *Mtb* thioredoxin Rv1471 as a protein subunit vaccine capable of inducing both innate immune memory in macrophages and adaptive immune memory in T lymphocytes [21]. However, the tuning effect on macrophage immune memory was only determined via *in vitro* cultured bone marrow-derived macrophages models, which poorly recapitulate lung-resident populations. AMs constitute the primary defense barrier against aerosolized *Mtb*, with their embryonic origin, self-renewal capacity, and strategic positioning making them ideal immunomodulation targets against respiratory pathogen such as *Mtb*. We therefore investigated Rv1471-induced trained immunity specifically in AMs.

Following inhalation, *Mtb* is initially phagocytosed by lung tissue-resident AMs, where incompetent AMs permit bacterial persistence while activated AMs control primary infection [36]. Once infection is established, *Mtb* subsequently disseminates from AMs and infects other macrophage populations including IMs and MDMs. These macrophages not only directly restrict bacterial growth but also process and present *Mtb*-derived antigens to CD4^+^/CD8^+^ T cells via dendritic cells, thereby facilitating adaptive immunity-mediated bacterial clearance. Thus, as the first line of defense against *Mtb* infection, AMs play a critical role in determining infection outcomes based on their functional states [37]. Notably, their embryonic origin enables long-term self-renewal independent of monocyte recruitment. Accumulating evidences indicate that trained AMs exhibit stable epigenetic modifications and sustained functional enhancement in murine models for at least three months [31,33,35], suggesting their potential as durable antimicrobial mediators. Given reports that chimpanzee adenoviral vectors induce autonomous AMs memory, we employed this delivery system.

Although multiple studies have shown that trained AMs upregulate phagocytic and scavenger receptors, inflammatory cytokines, and chemokines to control heterologous infections [20,31,33,35], *Mtb*-specific AMs training remains underexplored. By combining the trained immunity-inducing Rv1471 antigen with a chimpanzee adenoviral vector, we demonstrated that rAd-Rv1471 exhibits a potent capacity to induce trained immunity in AMs. Notably, we also generated a recombinant adenovirus expressing another *Mtb* antigen Rv2299c, rAd-Rv2299c, which failed to induce significant trained immunity in AMs (Figure S4), further confirming Rv1471’s specificity. AMs reside in oxygen-rich but glucose-poor alveolar environments, which necessitate oxidative phosphorylation rather than glycolysis as their primary metabolic pathway [38]. Consistent with the reports showing trained immunity induction is associated with enhanced glucose utilization and storage capacity in macrophages [20,30], including AMs [20], our analysis at both genetic (Figure 4) and metabolic levels (Figure S6D) revealed that rAd-Rv1471 significantly enhanced glycolysis and the TCA cycle in trained AMs compared to control groups, indicating metabolic reprogramming characteristic of trained immunity. Furthermore, we observed upregulated pathways for endocytosis, autophagy, mitophagy, ferroptosis, and apoptosis (Figure 4D), which might countering *Mtb’*s lysosomal fusion inhibition [39] and synergizing with known antimicrobial mechanisms [40–42].

In this proof-of-concept study, the strategy of utilizing the Rv1471 antigen delivered by a chimpanzee Ad vector to concurrently induce trained immunity in AMs and robust polyfunctional T-cell responses presents distinct advantages over other vaccine platforms. Ad have been reported to induce AMs trained immunity [20], a conclusion further supported by our data (Figures 2 and 3). Unlike adjuvanted protein subunit vaccines that primarily stimulate adaptive immunity through systemic delivery, the mucosal administration of our Ad vector directly targets lung-resident innate immune cells. This establishes a state of heightened alertness, enabling a faster response to subsequent *Mtb* exposure. Moreover, compared to mRNA or other nucleic acid-based platforms, viral vectors like the one employed here have been shown to more potently trigger the metabolic and epigenetic reprogramming characteristic of durable trained immunity in myeloid cells. Thus, chimpanzee Ad vector was chosen in this study.

Nevertheless, while the chimpanzee adenovirus platform proved effective in this proof-of-concept study, exploring Rv1471 within next-generation delivery systems remains crucial to fully delineate its potential and optimize a future TB vaccine. Alternative platforms, such as mRNA-lipid nanoparticle formulations, offer distinct advantages that could address specific limitations. mRNA-lipid nanoparticle vaccines enable rapid, scalable production and possess self-adjuvanting properties that may potentiate innate immune training. Critically, such platforms may help circumvent pre-existing adenoviral immunity, thereby promoting more equitable vaccine efficacy across diverse populations. Future comparative studies evaluating Rv1471 delivered via mRNA and other vector systems will be essential to identify the optimal strategy for inducing durable trained immunity in AMs and advancing TB vaccine development.

Consistent with prior reports [20], intranasal administration of the empty adenovirus vector itself induced a baseline level of trained immunity in AMs (Figures 2–4). Notably, the incorporation of the Rv1471 antigen into this vector substantially amplified the trained phenotype, as evidenced by enhanced cytokine production, elevated MHC-II expression, and superior control of intracellular mycobacterial growth, underscoring the antigen-specific contribution beyond the vector’s baseline effect, a point that could be further delineated using alternative Rv1471-expression platforms in future studies. It should be noted that Ad vector-trained AMs alone failed to confer protection against *Mtb* challenge (Figure 6), indicating that AMs activation is insufficient against sophisticated pathogens such as *Mtb*. In fact, the control of early *Mtb* infection requires the interplay between AMs and tissue-resident antigen-specific T-cell immune responses, as indicated by other reports: AMs could directly activate CD4^+^ T cells via MHC-II, triggering cytokines/chemokines secretion (TNF-α, IL-12, and CCL2/5/9) that recruits T cells to infection sites [43]; BCG-trained macrophages accelerate T cells activation and reduce bacterial loads [44], while IFN-γ from T cells amplifies macrophage function [20,45], highlighting the necessity of integrated immunity. Our data thus support a model of spatiotemporal synergy: intranasal rAd-Rv1471 vaccination rapidly establishes a state of trained alertness in AMs at the primary portal of infection, enhancing early bacterial containment. Concurrently, it seeds the antigen-specific, polyfunctional memory T cells in the lung. Upon *Mtb* challenge, these trained AMs can more efficiently process and present antigen, leading to the accelerated reactivation of the coexisting T-cell pools. This coordinated, two-layered response provides a plausible mechanism for the superior protection observed.

Given that neonatal BCG vaccination is already implemented in most high-TB-burden countries, the most strategic application of rAd-Rv1471 lies in its use as a heterologous mucosal booster. This approach directly addresses the key limitation of BCG, namely its variable efficacy in adolescents and adults, by specifically amplifying lung-resident trained immunity and T-cell memory. Therefore, rather than seeking to replace BCG, this platform is designed to enhance it, offering a targeted strategy to improve protection in high-risk populations. Furthermore, it establishes a versatile foundation for developing future multivalent booster vaccines.

As we have discussed in a recent review [46], one of the central challenges in TB vaccinology is that elevated immune readouts, such as cytokine levels or surface activation markers, do not consistently correlate with functional protection. This challenge is compounded by the ability of *Mtb* to induce non-protective or even decoy immune responses, which can mask a lack of effective immunity [47,48]. Furthermore, immune function might also be shaped by local tissue microenvironments. In non-granulomatous lung regions, AMs demonstrate upregulated expression of NF-κB and HIF-1α, along with increased production of proinflammatory mediators such as iNOS and ROS, which collectively contribute to effective bacterial clearance. Conversely, within fibrotic and hypoxic areas like the walls of tuberculous granulomas, AMs display a hypo-inflammatory phenotype and form immunologically privileged niches that support *Mtb* persistence [49]. This spatial regulation reflects our proposed “Yin-Yang” balance of trained immunity [16], wherein an early, robust response must be calibrated to avoid excessive inflammation and tissue damage during chronic infection. Therefore, an effective TB vaccine strategy must navigate this delicate balance by inducing potent immunity without precipitating harmful immunopathology. In this study, we propose our dual-targeting strategy might enhance bacterial control and antigen presentation within trained AMs, and then fostering integrated crosstalk with lung-resident T cells, to establish a more coordinated and efficacious immune defense at the mucosal portal of infection.

While the murine model provides a robust and controlled system for initial proof-of-concept, its limitations in recapitulating key aspects of human TB, such as granuloma maturation, cavitation, and chronic disease progression, must be acknowledged. To strengthen translational relevance, future studies should evaluate rAd-Rv1471 in non-human primate models, which more closely mirror human pulmonary immunopathology and allow for assessment of vaccine efficacy within structured granulomatous lesions. These models also enable the study of immune responses in anatomically and physiologically relevant mucosal compartments, offering insights into potential variations in vaccine uptake and local immunity. Following successful validation in non-human primate models, subsequent clinical translation should systematically account for population variability. Evaluating immunogenicity in Phase I/II trials that include participants from diverse genetic and geographic backgrounds will be crucial to de-risk the translational pathway and to inform the design of clinical trials targeting populations in whom BCG revaccination has shown limited benefit.

The current study has several limitations. First, although murine models are invaluable for initial proof-of-concept study, they cannot fully mirror the immunological and pathological complexity of human TB. Differences in lung anatomy, macrophage heterogeneity, and disease chronicity necessitate caution when extrapolating these findings directly to humans. Second, while rAd-Rv1471 potentially synergizes AMs and T-cell memory, this crosstalk requires validation through co-culture systems or adoptive transfer models. Third, although rAd-Rv1471 shows promising efficacy, combining it with immunodominant *Mtb* antigens (e.g. ESAT-6, TB10.4, and Ag85A/B) in multivalent vaccines might broaden protection by co-activating trained and adaptive immunity.

In conclusion, our study identifies rAd-Rv1471 as a dual-targeting vaccine integrating AMs training with polyfunctional T-cell responses, offering a synergistic strategy for TB control. These findings underscore the translational potential of incorporating AMs trained immunity into the rational design of next-generation vaccines.

## Materials and methods

### Recombinant adenovirus construction

The *Mtb Rv1471* gene and its upstream promoters were amplified by PCR and cloned into the shuttle plasmid pSRK17. The construct was then transformed into TOP10 competent cells, amplified, and the gene fragments were inserted into adenoviral backbone through homologous recombination. The recombinant constructs were transformed into XL10 competent cells for large-scale plasmid amplification. After endotoxin removal, the linearized recombinant adenoviral DNA were transfected into HEK-293A cells for viral packaging. The recombinant adenoviruses were purified by cesium chloride density gradient ultracentrifugation.

### Isolation and purification of AMs

Mice were euthanized, the trachea was exposed, and a cannula was inserted and secured. The lungs were lavaged three times with ice-cold RPMI-1640 (Hycolne) medium supplemented with 2% fetal bovine serum (FBS; Thermo Fisher Scientific) and 20 μg/ml gentamicin. BALF was collected, and red blood cells were lysed, and the remaining cells were incubated for 20 min with CD11c MicroBeads UltraPure (Miltenyi Biotec) at 4 °C. CD11c^+^ cells were isolated by magnetic-activated cell sorting (MACS) using positive selection according to the manufacturer’s protocol. All animal studies were approved by the Institutional Animal Care and Use Committee and were performed in accordance with the Laboratory Animal Ethical Board of Shanghai Public Health Clinical Center.

### *In vitro* AMs culture and training model

An *in vitro* expanded AMs trained immunity evaluation model was utilized [28,33]. Briefly, isolated AMs were cultured in RPMI-1640 medium supplemented with 10% FBS, 30 ng/ml granulocyte-macrophage colony-stimulating factor (GM-CSF; GenScript), and 20 μg/ml gentamicin. At 70-80% confluency, cells were detached with trypsin (Absin), counted, and seeded into 96-well plates at 5 × 10⁴ cells/well. After overnight adherence, the cells were treated for 24 h with PBS, Ad, or rAd-Rv1471, all at a MOI of 1. Supernatants were harvested, cells were washed and cultured with fresh culture medium for 5 days before downstream analysis.

### Enzyme-linked immunosorbent assay (ELISA)

ELISA assays for IL-1β, IL-6, and TNF-α were performed in accordance with the manufacturer’s instructions (Thermo Fisher Scientific). Absorbance was measured at 450 nm using a microplate reader.

### Protective efficacy of AMs against *in vitro* mycobacterial infection

AMs were obtained and rested following the previously described protocol. The cells were infected with BCG/*Mtb* at MOI of 5. The bacterial suspensions were centrifugated at 600× *g* for 5 min to enhance bacterium-cell contact, followed by incubation at 37°C for 1 h to phagocytosis. The cells were then washed to eliminate extracellular bacteria, and then were cultured in complete medium supplemented with 50 μg/ml gentamicin for 2 h. To assess phagocytic efficiency, a portion of cells was lysed with sterile water for 10 min, serially diluted and plated for colony-forming unit (CFU) enumeration. This time point, taken immediately after the 2 h gentamicin treatment, was designated “0 h”; the gentamicin step eliminates extracellular bacteria, so the CFU counts at 0 h reflects the initial burden of phagocytosed mycobacteria. The remaining cells were then cultured further in antibiotic-free complete medium at 37 °C and harvested at 24 h, 48 h, and 72 h for bacterial counting at each interval [21,50].

### Establishment of an *in vivo* trained immunity model in mice

Female specific pathogen-free BALB/c mice aged 6–8 weeks, purchased from Huachuang Xinuo Medical Technology Co., Ltd, were anesthetized with isoflurane, and PBS, 1 × 10⁸ plaque-forming units of Ad, or rAd-Rv1471 was administered via nasal instillation at a volume of 20 μl/mice. Mice were rested for 28 days post-administration to return to the baseline activation states.

### *In vivo* protective efficacy assessment against *Mtb* infection in mice

Trained mice were challenged with *Mtb* H37Rv via aerosol infection (∼100 CFU/mice). Four weeks post-infection, the animals were euthanized by cervical dislocation, and lungs and spleens were aseptically harvested, placed in 2 ml screw-cap tubes containing 1 ml PBS and 1.5 mm steel beads, and subsequently homogenized (MiniBeadbeater-16, BioSpec). Tissue homogenates were serially diluted in PBST-80 (PBS containing 0.05% Tween 80), and 100 μl aliquots were plated onto 7H11 agar plates (BD) supplemented with 10% OADC (oleic acid, albumin, dextrose and catalase medium; BD), 0.05% glycerol and an antibiotic mixture (40 U/ml polymyxin B, 4 mg/ml amphotericin, 50 mg/ml carbenicillin and 2 mg/ml trimethoprim) [51]. CFU were enumerated after 3 weeks.

### Preparation of single-cell suspension

In an aseptic environment, mouse spleens were harvested, and single-cell suspensions were prepared by grinding through gauze, followed by red blood cell lysis. Lung tissues were also harvested aseptically, minced, and digested in RPMI-1640 medium supplemented with 10% FBS, gentamicin (20 μg/ml), DNase I (200 μg/ml; Roche), and collagenase IV (1 mg/ml; Absin) at 37 °C for 45 min. The digested tissue suspension was then filtered through a 70 μm cell strainer (Thermo Fisher Scientific), and red blood cells were lysed. The resulting single-cell suspension from lung tissues was collected for further analysis.

### Hematoxylin and eosin (H&E) staining

Lung tissues were fixed in 4% paraformaldehyde (Absin) for 24 h in the dark, followed by paraffin embedding, sectioning, and H&E staining through a commercial service. Histopathological evaluation was carried out blindly by a qualified pathologist. The quantification of inflammatory infiltration was quantified by the TissueFAXS 200 system, and calculated as the percentage of hematoxylin-positive area to total tissue section.

### Flow cytometry

Cells were seeded into 96-well U-bottom plates at a density of 1∼2 × 10⁶ cells/well. For antigen stimulation, cells were treated with Rv1471 (10 μg/ml), HK *Mtb*-H37Rv (MOI = 50), or purified protein derivative (PPD; 20 μg/ml) overnight. GolgiStop (BD) and GolgiPlug (BD) was added 6 h prior to harvest. Cells were washed with cold PBS, stained with viability dye (BD) at room temperature for 15 min. After washing, the cells were subjected to surface staining with fluorescently labelled antibodies at 4°C for 30 min in the dark. Cells were then fixed and permeabilized using a Fixation/Permeabilization Kit (BD) or Transcription Factor Buffer Set (100 μl/well, 4°C, 30 min; BD), followed by intracellular and nuclear antibody staining at 4°C for 30 min in the dark. After resuspension in staining buffer and filtration through a cell strainer, samples were analyzed using an LSRFortessa flow cytometer (BD). Data were processed and analyzed with FlowJo v10 software.

### Glycolytic rate assay

All experiments were conducted using instruments and reagents provided by Agilent Technologies. AMs from trained mice (8 × 10⁵ cells/well) were seeded in Seahorse XF24 plates and cultured overnight. The Seahorse probe was hydrated one day prior to the assay. Assay medium rotenone (R/A) and 2-deoxyglucose stock solutions were prepared according to manufacturer's instructions. Following microscopic confirmation of uniform cell density, medium was replaced with pre-warmed assay medium and the plates were equilibrated at 37°C in a CO₂-free incubator for 1 h. Compounds were prepared in accordance with the manufacturer's instructions, loaded into the sensor cartridges, and calibrated using WAVE software prior to initiation of the assay. Data acquisition and analysis were performed using the same software platform.

### qPCR

Cells were lysed in TRIzol (Takara), and chloroform (one-fifth of the TRIzol volume) was subsequently added. Following incubation and centrifugation, the aqueous phase was collected and RNA was precipitated with isopropanol. The RNA pellet was washed with 75% ethanol, air-dried, and resuspended in RNase-free water. RNA concentration and purity were assessed using a NanoDrop 2000 spectrophotometer (Thermo Fisher Scientific). cDNA was synthesized through reverse transcription using the HiScript III RT SuperMix reagent (Vazyme), following the manufacturer's recommended protocol. Quantitative PCR was carried out using the PowerUP SYBR Green Master Mix (Promega) on a Quant Studio 3 Real-Time PCR System (Thermo Fisher Scientific) under standard cycling conditions. The primer sequences used in this study are listed in Table 1.

**Table 1.** The qPCR primers.

Genes	Forward Primers	Reverse Primers
*Tnfα*	TAGCCCACGTCGTAGCAAAC	TGTCTTTGAGATCCATGCCGT
*Il6*	GGGACTGATGCTGGTGACAA	ACAGGTCTGTTGGGAGTGGT
*Il1b*	TGCCACCTTTTGACAGTGATG	TGATGTGCTGCTGCGAGATT
*Nfkb*	AAAGGCCCCCTTGCAACAGA	CAGGTTTGCAAAGCCAACCAC
*Mtor*	GCATAACAGATCCTGACCCTGAT	TTCAGAGCCACAAACAGAGC
*Akt1*	CCGCCTGATCAAGTTCTCCT	GATGATCCATGCGGGGCTT
*Hif1a*	CCTGTAAGCAAGGAGCCAGA	ACTGTCTAGACCACCGGCAT
*Nod1*	TTAAGGGTGAAGCCAAAGGGTC	AATGGGGTGCCTTCCATCTC

### Smart-RNA-Seq

The trained AMs were mixed with lysis buffer and sent to Astrocyte Technology Company for reverse transcription to synthesize cDNA. Following quality control, the purified cDNA was used for fragmentation via the TN5 reaction. Library construction was carried out using amplification reagents and sequencing adapters. Sequencing was conducted on the Illumina Xplus platform. The quality of the raw sequencing data was assessed using FastQC, followed by alignment of the filtered data to the reference genome with HISAT2. Gene expression levels were then quantified annormalized using Stringtie. Following the removal of gene expression data containing zero values or missing entries, differential gene expression analysis was carried out using DESeq2. Functional enrichment analysis was then performed using the “org.Mm.eg.db” annotation database and the “clusterProfiler” package. The threshold for identifying differentially expressed genes was set as |log_2_FC| > 1 and an adjusted *P*-value < 0.05.

### Statistical analysis

Statistical analysis was performed using GraphPad Prism software (version 10.1.2). All data are expressed as the mean ± standard deviation (SD). Comparisons among multiple groups were performed using one-way analysis of variance (ANOVA) followed by Tukey’s post-hoc test, or two-way ANOVA followed by Dunnett’s multiple comparison test.

## Author contributions

Conceptualization: Z.H. and X.F. Methodology: H.W., Y.Z., J.L., J.W., S.H., S.X. and X.H. Data analysis: H.W., J.W., and Z.H. Original writing: H.W., and Z.H. Review and editing: Z.H., J.W., and X. F. Supervision and funding: Z.H., X.F., and J.W.

## Supplementary Material

Table S1.csv

260107_Supplement_materials_final-clean.docx

## Data Availability

The RNA-seq data have been deposited in the NCBI Sequence Read Archive (SRA) under the accession number PRJNA1392094 and are publicly accessible.
