# Supplementary material for: An Rv1471-expressing chimpanzee adenovirus vaccine confers protection against tuberculosis by inducing alveolar macrophage trained immunity and polyfunctional T-cell responses
Source: Emerg Microbes Infect. 2026 Feb 24;15(1):2637292. doi: 10.1080/22221751.2026.2637292 (PMC12978187; doi:10.1080/22221751.2026.2637292)
Supplement: 260107_Supplement_materials_final-clean.docx [file TEMI_A_2637292_SM7490.docx]

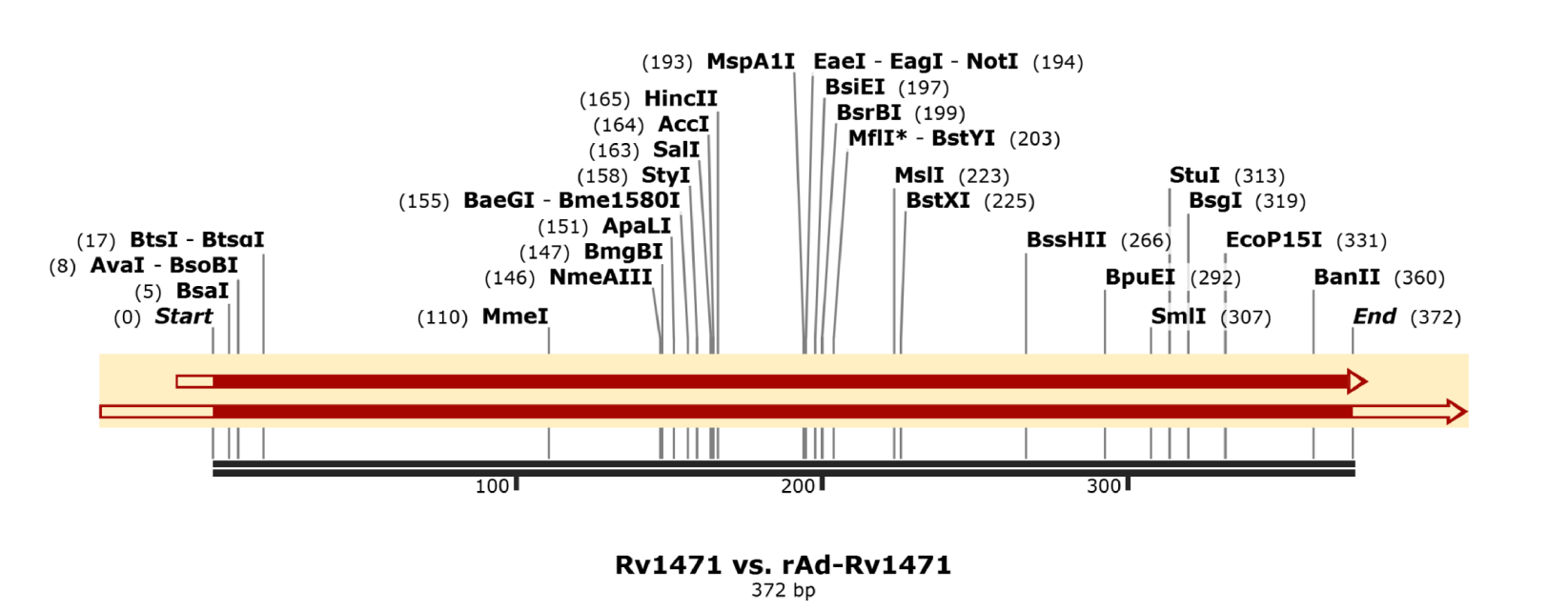


**Figure S1. Construction and validation of rAd-Rv1471.** Alignment of Sanger sequencing results of rAd-Rv1471 with the reference *Rv1471* sequence from NCBI.


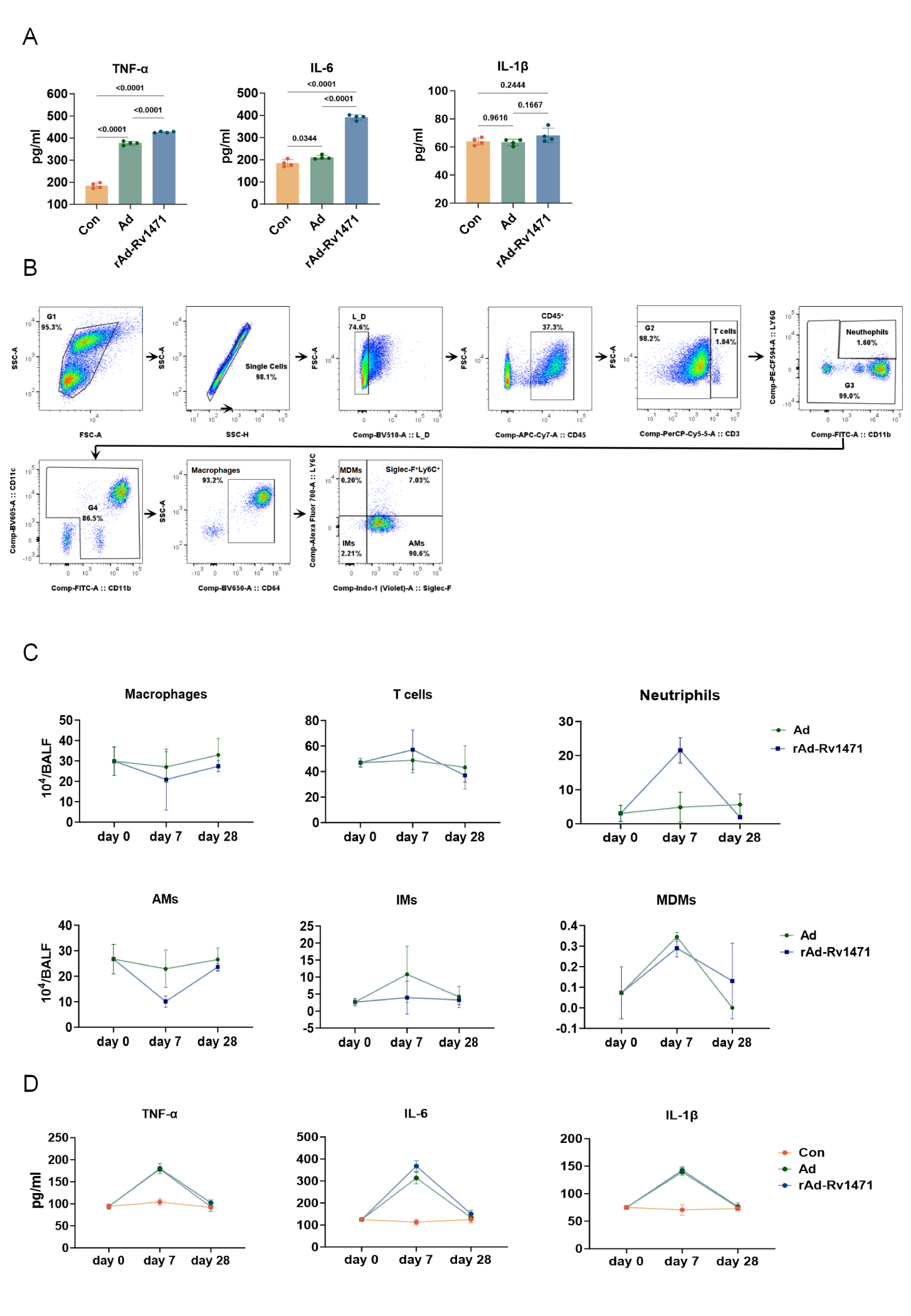


**Figure S2. The effects of intranasal rAd-Rv1471 on the cell population in BLAF and proinflammatory cytokines secretion in AMs. (A)** Levels of TNF-α, IL-6, and IL-1β in the culture supernatants measured by ELISA following LPS stimulation (n = 4; one-way ANOVA). **(B)** Schematic of BALF flow cytometric gate strategy. **(C)** Cellular composition in BALF analyzed by flow cytometry (n = 4; two-way ANOVA). **(D)** Serum levels of TNF-α, IL-6, and IL-1β measured by ELISA (n = 4; two-way ANOVA). **Data represent two independent representative experiments and are presented as mean ± SD.**

**
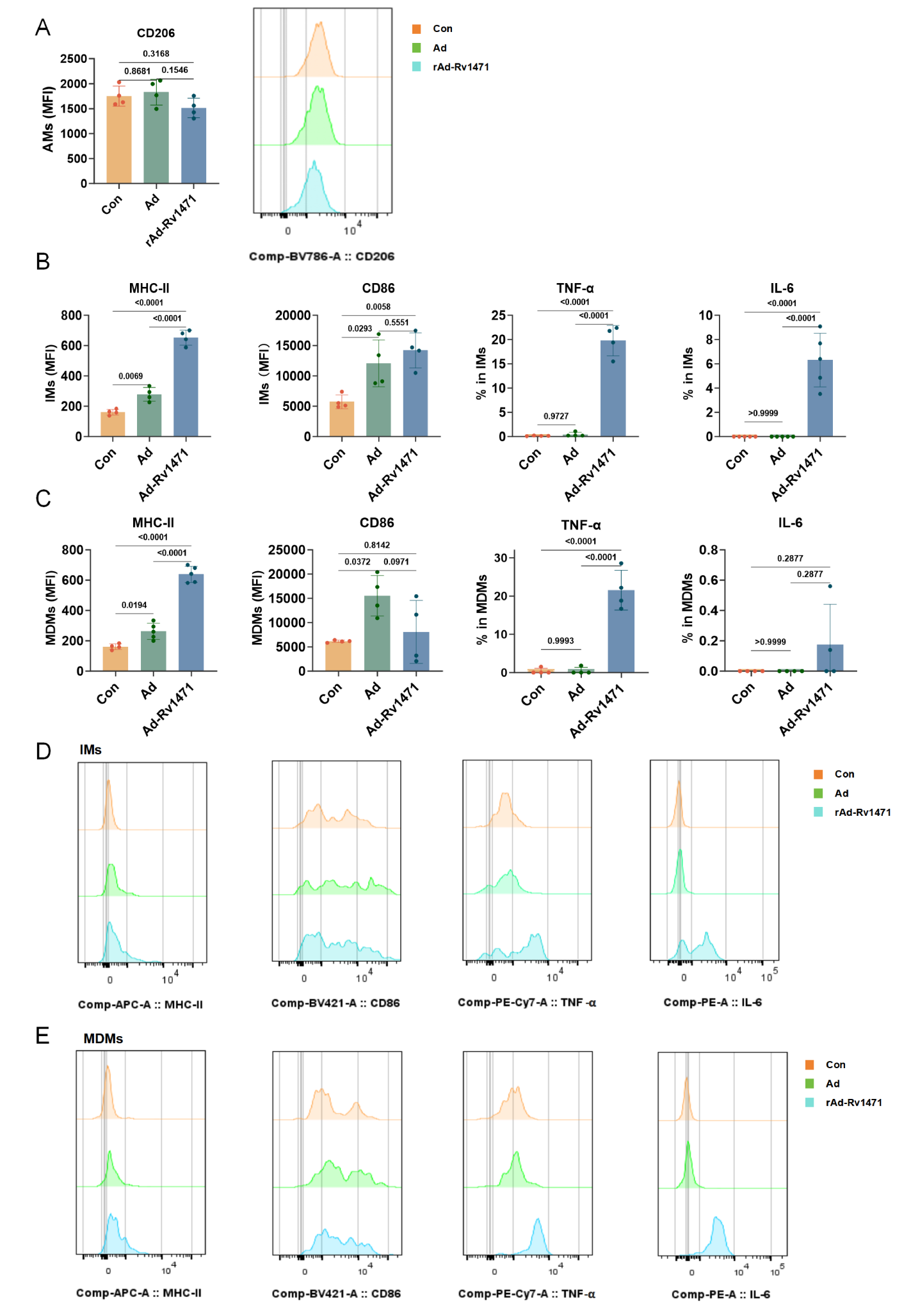
**

**Figure S3. Activation marker expression and inflammatory cytokines secretion of myeloid cells following intranasal rAd-Rv1471 training.** **(A)** MFI of CD206 and representative flow cytometric plots on AMs (n = 4; one-way ANOVA). **(B-D)** MFI of MHC II/CD86 and secretion levels of TNF-α/IL-6 in IMs **(B)** and MDMs **(C)** (n = 4; one-way ANOVA), along with representative flow cytometric plots shown in **(D-E)**. Cell gating was based on the strategy outlined in Fig. S2B. **Data represent two independent representative experiments and are presented as mean ± SD.**

**
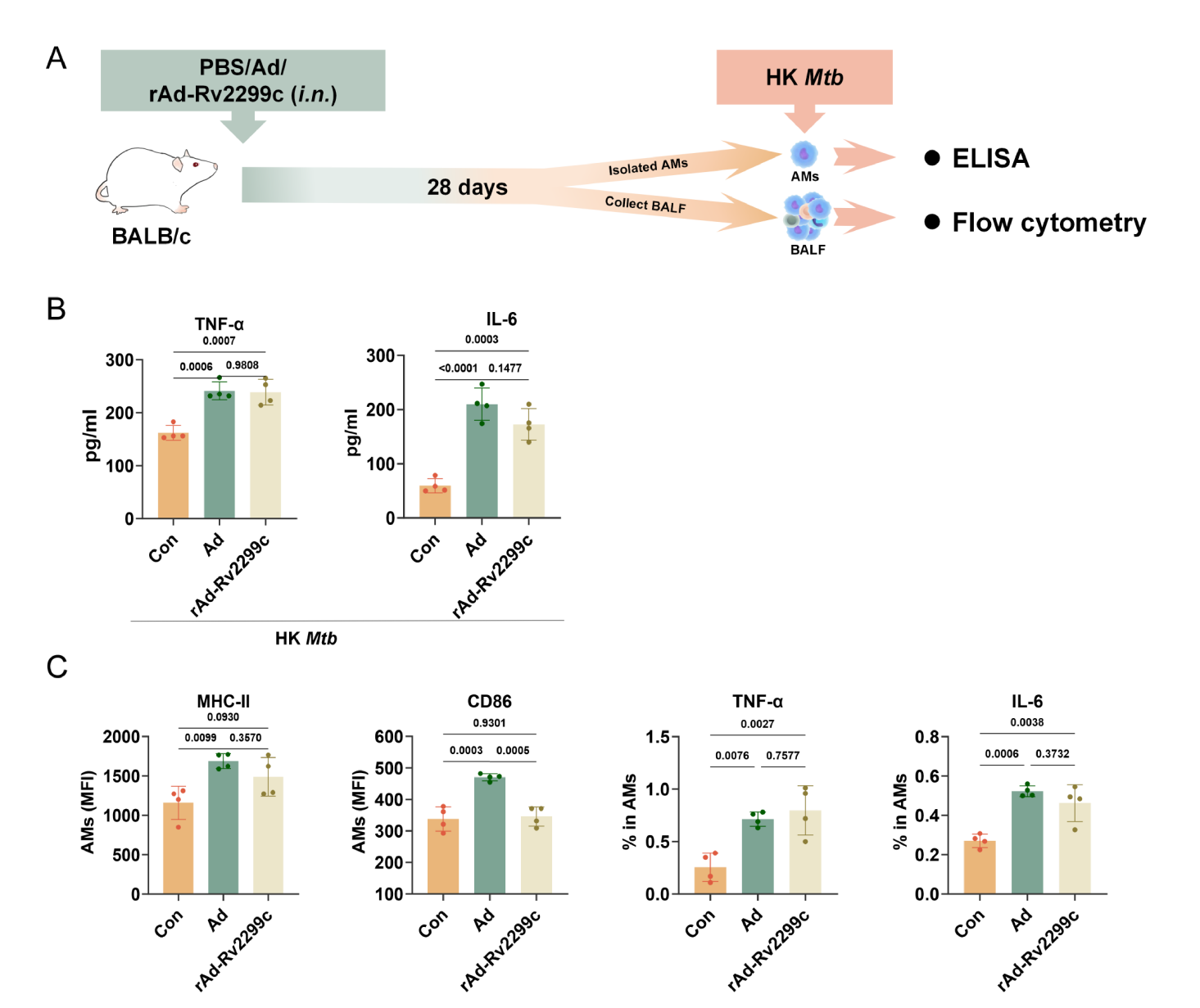
**

**Figure S4. rAd-Rv2299c failed to induce trained immunity in AMs. (A)** **Schematic representation of the mouse trained immunity evaluation model.** BALF was collected 28 days after *in vivo* training with PBS, Ad, or rAd-Rv2299c for subsequent immunological testing. **(B)** ELISA was performed to quantify the levels of secreted TNF-α and IL-6 in the culture supernatants following HK *Mtb* stimulation. **(C)** MFI of MHC II/CD86 and secretion levels of TNF-α/IL-6 in AMs **(n = 4; one-way ANOVA)**. **Data represent two independent representative experiments and are presented as mean ± SD.**


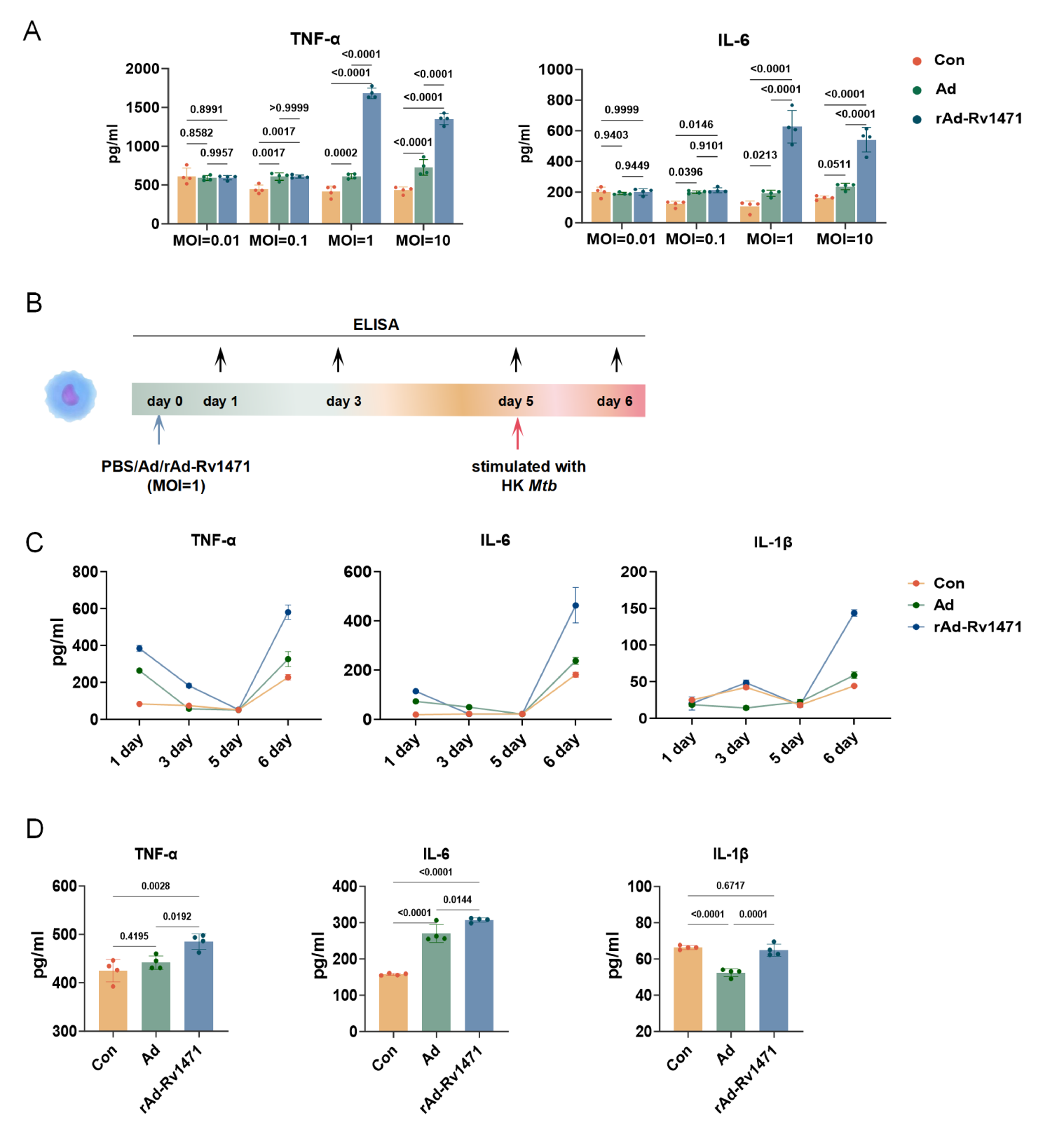


**Figure S5. The kinetics of proinflammatory cytokines production after rAd-Rv1471 training.** (A) AMs were trained with Ad, rAd-Rv1471, or PBS at the indicated multiplicities of infection (MOIs of 0.01, 0.1, 1, and 10). After a 5-day rest period, cells were challenged with LPS, and the secretion levels of TNF-α and IL-6 in the culture supernatants were measured by ELISA. **(B)** **Schematic representation of the *in vitro* AMs training model.** **(C)** The secretion levels of TNF-α, IL-6, and IL-1β measured by ELISA in supernatant on days 1, 3, and 5 following PBS, Ad, or rAd-Rv1471 stimulation, as well as 1 day after HK *Mtb* re-stimulation (6 day) of sorted AMs (n = 4; two-way ANOVA). **(D)** **ELISA quantification of TNF-α, IL-6, and IL-1β in the culture supernatants following LPS re-stimulation (n = 4; one-way ANOVA). Data represent two independent representative experiments and are presented as mean ± SD.**


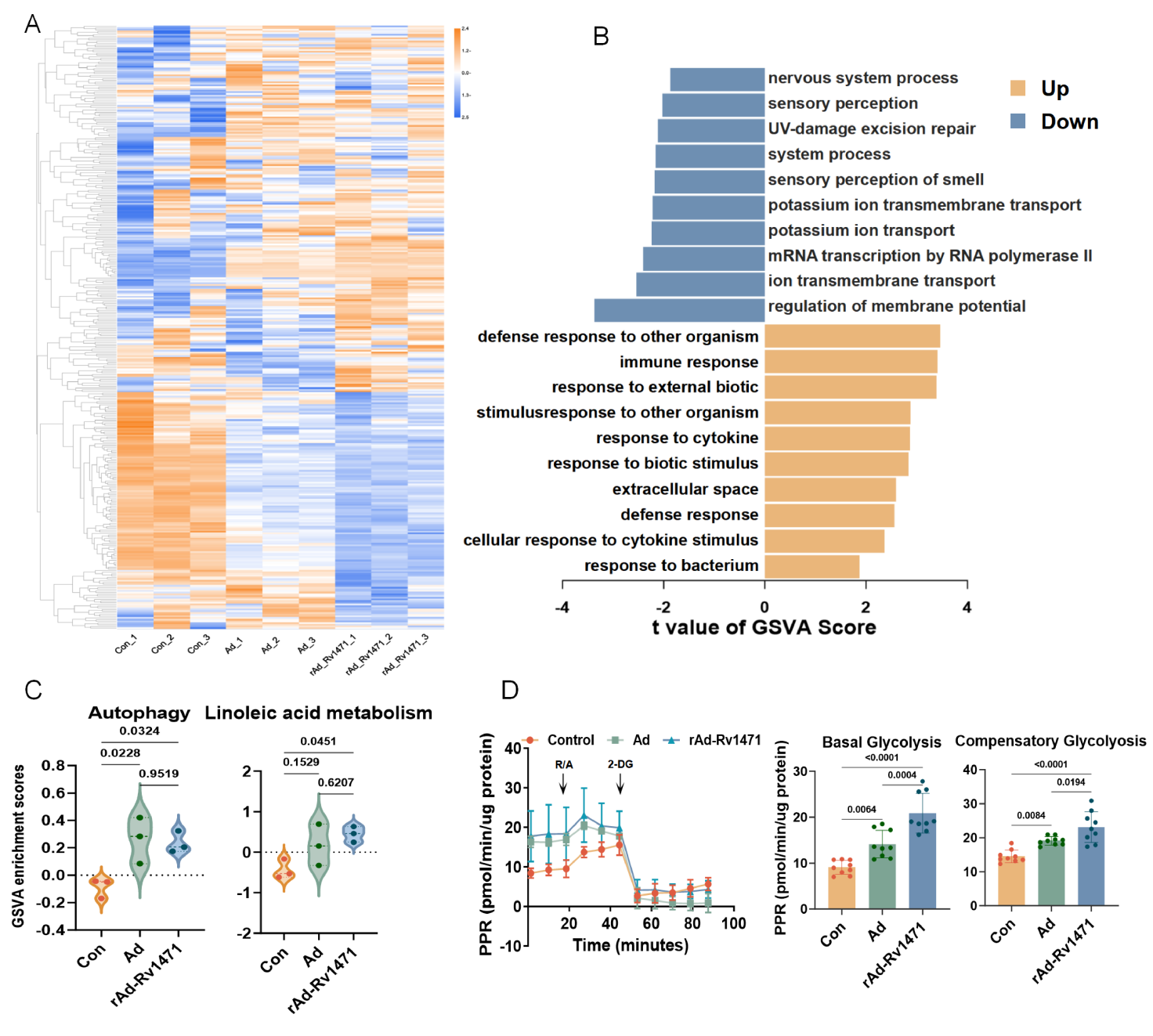


**Figure S6. Transcriptional profiles underlie rAd-Rv1471-induced trained immunity. (A)** Heatmap of all differentially expressed genes. **(B)** Top 10 significantly upregulated and downregulated pathways based on GSVA enrichment analysis. **(C)** Violin plots of GSVA enrichment scores for autophagy and linoleic acid metabolism (n = 3; one-way ANOVA). **(D)** The left panel shows the proton release rate (PPR) of AMs after 28 days of training, and the right panel shows the corresponding basal glycolysis and compensatory glycolysis analysis, the data are representative of three independent biological replicates, with each sample assayed in technical triplicate (n = 9; one-way ANOVA). **Data represent two independent representative experiments and are presented as mean ± SD.**


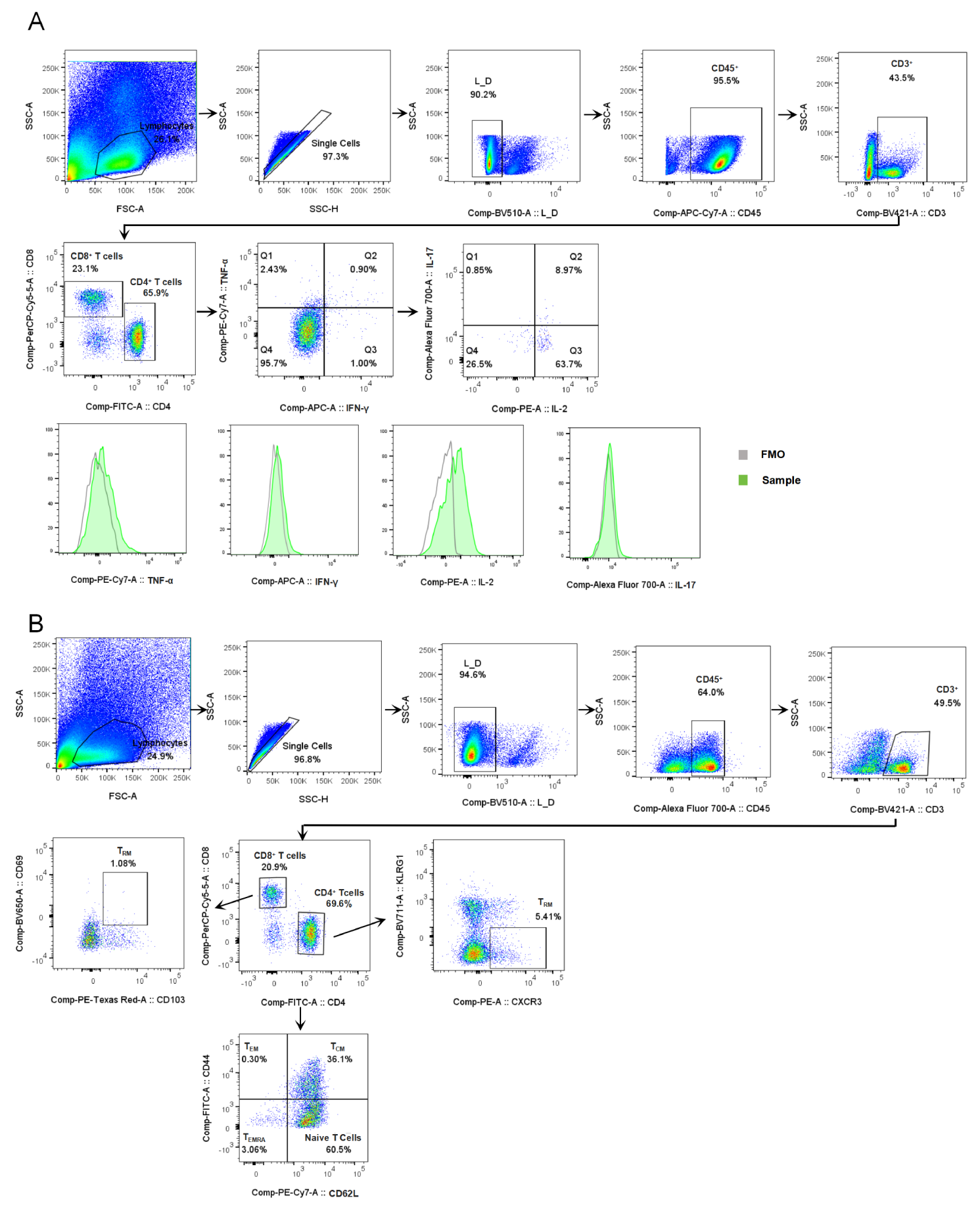


**Figure S7. Gating strategy for T-cell analysis. (A)** Cytokine detection gating in CD4^+^ and CD8^+^ T cells. **(B)** Gating strategy for naïve T cells, T_CM_, T_EM_, T_EMRA_, and T_RM_ subsets within CD4^+^ and CD8^+^ T cells.


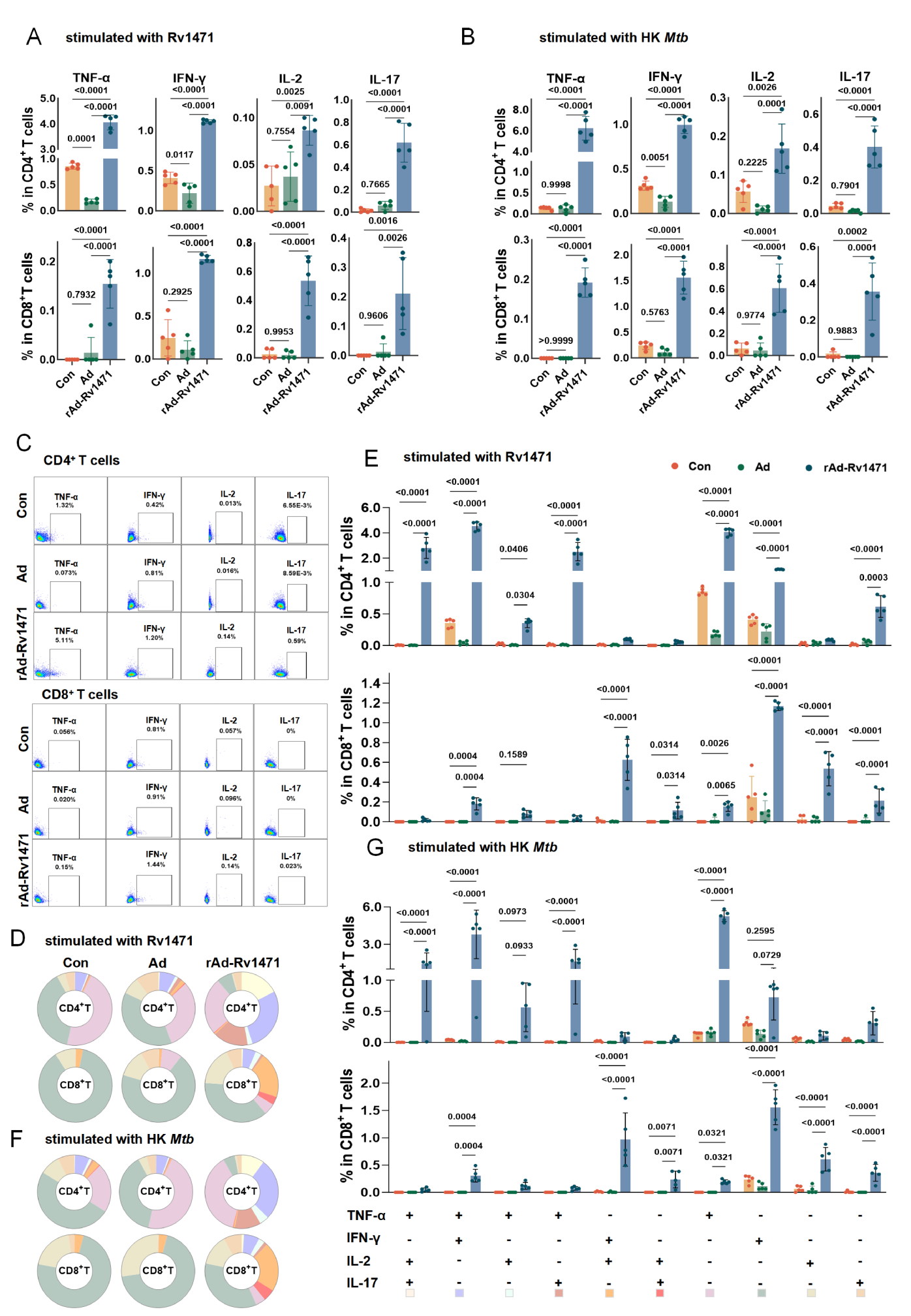


**Figure S8. rAd-Rv1471 induces robust antigen-specific T-cell immune responses. (A-B)** **Flow cytometric analysis of the T-cell immune response in the spleens 4 weeks post-trained with PBS, Ad, or rAd-Rv1471, following stimulation with Rv1471** **(A)** or HK *Mtb* **(B) (n = 5; one-way ANOVA)**. **(C)** **Representative flow cytometric plots depicting the expression of four inflammatory cytokines.** **Proportions (D) and frequencies (E) of Rv1471-specific polyfunctional CD4^+^ and CD8^+^ T cells; as well as proportions (F) and frequencies (G) of polyfunctional CD4^+^ and CD8^+^ T-cell immune responses following HK *Mtb* stimulation (n = 5; two-way ANOVA). Data represent two independent representative experiments and are presented as mean ± SD.**
